# Total versus near-total thyroidectomy in Graves’ disease and their outcome on postoperative transient hypoparathyroidism: study protocol for a randomized controlled trial?

**DOI:** 10.1186/1745-6215-13-234

**Published:** 2012-12-06

**Authors:** Katja Maschuw, Katja Schlosser, Dirk Lubbe, Christoph Nies, Detlef Klaus Bartsch

**Affiliations:** 1 Department of Visceral, Thoracic and Vascular Surgery, University Hospital Giessen and Marburg GmbH Location Marburg, Baldingerstrasse, Marburg, D-35043, Germany; 2 Coordination Center for Clinical Trials-KKS, Philipps-University Marburg, Karl-von-Frisch-Strasse 4, Marburg, D-35043, Germany; 3 Department of General and Visceral Surgery Marien Hospital, Bischofsstrasse 1, Osnabrueck, D-49074, Germany

**Keywords:** Graves’ disease, Transient postoperative hypoparathyroidism, Total thyroidectomy, Near-total thyroidectomy

## Abstract

**Background:**

Graves’ disease is an independent risk factor for transient postoperative hypoparathyroidism. Besides the disease itself, preparation techniques are influential. Transient postoperative hypoparathyroidism has severe consequences for patients’ physical and psychological state. It can be life threatening during the acute phase and may impair patients’ health, psyche and quality of life thereafter. For the surgical therapy of Graves’ disease, total thyroidectomy is recommended according to the national S2-guideline. The evidence- based on a metaanalysis- is criticized by the Cochrane diagnostic review commentary for substantial methodological deficits. Two randomized controlled trials lead to the hypothesis that a near-total resection with bilateral remnants of ≤ 1g on each side compared to total thyroidectomy will significantly reduce the occurrence of transient postoperative hypoparathyroidism with equal therapeutic safety.

**Methods/Design:**

Patients with Graves’ disease indicated for definite surgery are eligible for the trial. Trial-specific exclusion criteria are: conservative treatment, malignancy, previous thyroid surgery and coincident hypoparathyroidism. The trial is created for therapeutic purpose through process innovation. It is designed as a prospective randomized controlled patient and observer blinded multicentered trial in a parallel design including an active comparator and an intervention group. The intervention addresses the surgical procedure: near-total thyroidectomy leaving bilateral remnants of ≤ 1g on each side in the intervention group and total thyroidectomy in the control group. The occurrence of transient postoperative hypoparathyroidism is defined as primary endpoint. Secondary endpoints are: reoperations due to bleeding, recurrent laryngeal nerve palsy, permanent hypoparathyroidism, recurrent disease, changes of endocrine orbitopathy and quality of life within a one-year follow-up period. The primary efficacy analysis follows the intention-to-treat principle. A binary logistic regression model will be applied. Complications and serious adverse events will be descriptively analyzed.

**Discussion:**

The trail is expected to balance out the shortcomings of the current evidence. It will define the surgical gold standard for the surgical therapy of Graves’ disease. Patients’ safety and quality of life are assumed to be enhanced. Therapy costs are likely to be reduced and health care optimized. The conduction of the trial is feasible through the engagement and commitment of the German association of endocrine surgeons and the National Network for Surgical Trials.

**Trial registration:**

German clinical trials register (DRKS) DRKS00004161

## Background

Graves’ disease has been identified as an independent risk factor for transient postoperative hypoparathyroidism in retrospective trials. It was confirmed by a comprehensive prospective study including 5,846 consecutive patients in 2003 [1]. In the only existing randomized controlled trial, transient hypoparathyroidism is reported with an incidence of 28% after total thyroidectomy for Graves’ disease [2]. The estimated prevalence is calculated with 2,000 patients per year according to the available data [2-4]. The underlying causes are complex. Preparation techniques, especially the extent of resection seem to be more influential than inadvertent parathyroidectomy [1,5]. Postoperative hypoparathyroidism seriously affects physical health, wellbeing and quality of life. Acute hypocalcaemia can cause cramping, bronchial spasms, cardiac and digestive dysfunction and can therefore be life threatening. Long-term effects result in cataract, conjunctivitis, pruritus, osteopenia, calcifications of the basal ganglia (Fahr’s syndrome), neurological and psychic impairments. Despite calcium and vitamin D substitution, wellbeing is predominantly impaired by anxious and phobic psychological disorders [6]. Care and treatment costs as well as the status of employee’s illness are therefore of economic relevance.

At present, total thyroidectomy is favored for definite surgery in Graves’ disease instead of subtotal resection, according to the national S2-guideline issued by the Work Group of the Scientific Medical Professional Societies (AWMF) [7]. The current evidence supporting a total thyroidectomy in Graves’ disease is based on two randomized controlled trials [2,8], one meta-analysis [9] and four retrospective trials [10-13]. All studies evaluate the outcome after total versus subtotal thyroidectomy, in relation to the incidence of recurrent disease. The meta-analysis, cited in the national S2-guideline, displayed a zero-recurrence rate and an equal risk of method-associated complications [9]. However, the corresponding Cochrane diagnostic review commentary emphasizes group inhomogeneity as a substantial methodological deficit. Four retrospective trials [10-13] demonstrated a significantly lower incidence of transient hypoparathyroidism after subtotal, compared to total resections, while the incidence of recurrent disease differed according to variable sizes of the remnants (mean 6.1 g, range 1 to 12 g) and follow-up periods. Among these four trials only one clearly defined the size of the remnant with a unilateral residual thyroid tissue of ≤ 2 g when a subtotal resection was performed [13]. With this measure, the incidence of hypoparathyroidism significantly decreased, while the incidence of recurrent disease remained unaltered within a median follow-up of 6.7 years. The recurrence rate of 0.5% in this study was attributable to inadvertently spared pyramidal lobes in all patients as the result of a systematic default. Two prospective randomized controlled trials by Witte *et al*., 2000 [2] and Barczynski *et al*., 2012 [8] again aimed at recurrence of Graves’ disease as the primary endpoint, addressing different remaining tissue volumes and resection techniques. The trial by Witte *et al*. compared two interventional arms: one arm comprised a unilateral total resection and a contralateral subtotal resection with a remaining remnant of ≤ 4 g; the second arm comprised a bilateral subtotal resection with a remaining remnant of ≤ 4 g in total to the control arm total thyroidectomy. Barczynski *et al*. considered bilateral remnants of approximately 2 g on each side. Both trials displayed a significantly lower incidence of transient hypoparathyroidism in the intervention groups. While the incidence of recurrent disease was not significant, clinically not relevant, and detectable in all affected patients six months after surgery, after a follow up of 18 to 58 months in the trial by Witte *et al*., Barczynski *et al*. reported significantly higher relapse rates within 60 months follow up. Endocrine orbitopathy did not worsen after subtotal resection in both trials. So far, the evidence from the prospective randomized controlled trials seems contradictory regarding the incidence of clinically relevant recurrent disease. Moreover, the S2-recommendation lacks appropriate evidence in view of comorbidities. According to our knowledge, the incidence of transient hypoparathyroidism has never been valued as primary endpoint. Current data suggest a relevant reduction of the incidence of transient hypoparathyroidism after a near-total resection if defined remnants of ≤ 1 g on each side are left around the posterior suspensory ligament of Berry (Ligamentum thryoihyoideum laterale). The resection will be safe compared to standard total thyroidectomy regarding recurrent disease, endocrine orbitopathy and method associated complications [2,9,13]. A prospective randomized controlled trial is necessary to define the gold standard resection for the surgical therapy of Graves’ disease in view of patients’ safety and quality of health care.

## Methods/Design

### Eligibility

Patients with Graves’ disease scheduled for definite surgery are eligible for the present study. Indications for definite surgery follow those of the national S2-guideline [7], in detail a high risk of recurrence (age below 40 years, male gender, thyroidea receptor anti-body (TRAK) serum levels > 10 U/l six months after conservative treatment, thyroid volume > 40 ml), persistent, recurrent or severe hyperthyroidism after primarily conservative therapy, thyroid growth, endocrine orbitopathy and refused radioactive iodine therapy. After a screening visit patients will be allocated into the trial according to the inclusion and exclusion criteria shown below.

### Inclusion criteria

Key inclusion criteria are: indication for definite surgery according to the national guideline, no history of previous thyroid and/or parathyroid surgery, normal vocal cord function, age greater than 18 years, expectancy of life greater than 12 months, and provision of informed consent.

### Exclusion criteria

Exclusion criteria are: eligibility for conservative treatment according to the national guideline, (suspected) malignancy, coincident hyperparathyroidism, and neurophysiological deficiencies.

### Intervention and control

The study is designed as a prospective, randomized, controlled, observer- and patient-blinded, multicentered clinical trial in a parallel design, including an intervention group and an active comparator. The intervention addresses the surgical procedure. It is defined as near-total thyroidectomy with bilateral remnants ≤ 1 g on each side for inclusion in the intervention group. After ligation of the upper pole vessels, and identification and preservation of the recurrent laryngeal nerve and parathyroid glands, the thyroid is removed from the lateral tracheal wall up to the superior suspensory ligament of Berry (ligamentum thyreohyoideum laterale). Here, the thyroid is resected leaving a piece of tissue assessed by ruler measurement accounting for 0.5 × 0.5 × 0.5 cm in size at most (≤ 1 g). After resection, the remnant *in situ* is photo documented. To ensure adequate sizes of the thyroid remnants in the intervention group, after removal of the specimen a representative piece of thyroid tissue (≤ 1 g) is excised, photo documented on a clean towel next to the ruler and weighed. This tissue fragment is later added to the specimen and both are sent for histopathology. Surgery in the control group comprises a total thyroidectomy where the whole thyroid gland must be removed [7]. The surgical sites of each side will be photo documented after resection.

### Surgical methods

Cervical approach, preparation, resection approach and vessel control are not limited per protocol in either group. The only binding preparation principles in either group are visualization, monitoring and protection of the recurrent laryngeal nerve, as well as the identification and protection of the parathyroid glands following the national guideline [7]. Neuromonitoring is performed and recorded before and after resection via the vagus nerve. If unforeseen multinodular changes, suspected malignancy, or frozen-section assured malignancy requires total thyroidectomy in the intervention group, or even additional lymphadenectomy in either group, the reason will be documented on the case report form. Required by the intention-to-treat principle, patients must be followed up in the group to which they are randomized.

### Definition of endpoints and outcome measures

#### Primary endpoint

The incidence of transient hypoparathyroidism is defined as the primary endpoint. Transient hypoparathyroidism is defined as an inadequate parathyroid function not exceeding six months after surgery [14]. Serum calcium (Ca) and parathyroid hormone (PTH) levels and substitution medication to achieve normocalcaemia are used to assess hypoparathyroidism. According to the best of our knowledge, there is no evidence- or consensus-based definition of postoperative hypoparathyroidism based on calcium and PTH cutoff levels. In line with most experts, we define postoperative hypoparathyroidism as calcium and PTH levels below the normal range (Ca ≤ 2.1 mmol/l, PTH < 11 ng/l). Postoperative hypoparathyroidism is considered to be symptomatic in the case of at least one of the following symptoms: carpo-pedal paresthesia, numbness or spasms, and anxiety. Application type, form and dosage of Ca and vitamin D substitution therapy to achieve normocalcaemia (Ca = 2.2 to 2.7 mmol/l) are registered. Postoperative hypoparathyroidism is defined transient if calcium and vitamin-D substitution therapy to achieve normocalcaemia are required for less than six months after surgery. The parameters are recorded before surgery, at the day of discharge, 6 weeks, 6 months and 12 months after surgery.

#### Secondary endpoints

Secondary endpoints comprise the incidences of permanent hypoparathyroidism, recurrent Graves’ disease, temporary and permanent recurrent laryngeal nerve palsy and reoperations due to bleeding, and the number of inadvertently removed parathyroid glands, as well as changes of endocrine orbitopathies and quality of life. Permanent hypoparythroidism is defined as persisting inadequate parathyroid function exceeding six months after surgery [15,16]. The incidence of recurrent Graves’ disease is assessed through laboratory thyroid function tests as thyroid stimulating hormone (TSH) < 0.34 U/l, free triiodinethyronine (fT3) > 6.5 pmol/l, thyroxine (fT4) > 21 pmol/l, and TRAK > 1.75 U/l, at 6 weeks, 6 months and 12 months after surgery, with respect to concomitant substitution or thyrostatic medications to achieve euthyrosis, determined by laboratory thyroid function tests (TSH, fT3, fT4). Cervical ultrasound follows detection of elevated antibody levels to screen for recurrence. Reoperations are reported as serious adverse events. Incidences of temporary and permanent recurrent laryngeal nerve palsies are assessed through laryngoscopy on the day of discharge and in the case of pathological findings 6 weeks, 6 months and 12 months after surgery. The number of inadvertently removed parathyroid glands will be counted from histopathology reports at the day of discharge. Changes in endocrine orbitopathy are measured using the validated clinical eyelid, exophthalmos eye muscle, optic nerve (LEMO) score [17] before and 12 months after surgery. Changes in quality of life are scored before surgery and 6 weeks thereafter using the validated multipurpose questionnaire, the clinical studies 36-item medical outcomes study short-form general health survey (SF36) [18].

### Randomization, allocation concealment and blinding

A central randomization will be performed, stratified by the performing surgeons. Only surgeons who have performed at least 100 thyroidectomies working at a volume center accounting for more than 100 thyroidectomies per year will participate. A random sequence will be generated through computerized random assignment and concealed allocation. Patients and the observer are blinded to the applied surgical intervention. Intraoperative randomization will be performed. The surgeon will be unblinded after the skin incision, and the patient and the observer will be blinded throughout the whole study period. All assessments will be performed by personnel who are blinded to the study arms for the minimization of measurement bias. Data storage and analyses will be performed externally.

### Sample size

The trial is designed on the basis of a critical appraisal of current evidence. According to the only existing randomized controlled trial by Witte *et al*. [1], a significant reduction in the incidence of transient hypoparathyroidism from 28% in the total thyroidectomy group to 12% in the near-total thyroidectomy group is suspected. Using a level of significance of 5% and a power of 80%, 97 patients per group are needed to detect the pre-specific difference when applying a chi square test (2-sided) without Yates's correction for continuity. With a dropout rate of 5% between obtaining informed consent and randomization, a total sample of 206 patients need to be recruited to the trial. Analysis of the primary endpoint will be performed by an adjusted logistic regression model. Therefore, it is to be expected that including covariates of prognostic importance in the logistic regression model will increase the power as compared to the chi square test. However, since the impact of the covariates cannot be determined a priori, a potentially liberal sample size estimation for the chi square test seems appropriate.

### Analysis

The primary hypothesis is whether the rate of transient hypoparathyroidism within 6 months after surgery is lower in patients after near-total compared to total thyroidectomy. A binary logistic regression model will be applied for the intervention comparison of the rates, adjusting for age, gender, hyperthyrosis and endocrine orbitopathy as additive covariates. The dropout rate can be assumed to be small, and therefore, few missing values are expected with respect to the primary endpoint. If missing values occur their reason of omission will be explored. Depending on the properties and magnitude of missing values, an appropriate estimation will be specified before the analysis of the primary endpoint. Concerning other secondary endpoints, exploratory data analysis will be performed calculating appropriate summary measures for the empirical distribution as well as calculation of descriptive two-sided 95% confidence intervals and *P*-values. Sensitivity analyses will be conducted for the per-protocol population as well as for appropriate subgroups. The safety analysis includes calculation and comparison of frequencies and rates of adverse and serious adverse events. Furthermore, statistical methods are used to assess the quality of data and the homogeneity of intervention groups.

## Discussion

The S2-recommendation of total thyroidectomy for Graves’ disease based on the national guideline lacks appropriate evidence in view of morbidity. Total thyroidectomy compared to subtotal or near-total resection predisposes patients to a relevantly higher risk for transient postoperative hypoparathyroidism. The consequences of transient postoperative hypoparathyroidism can be severe and life threatening during the acute phase and may impair patient’s psychological state and quality of life thereafter. According to the existing evidence, near-total thyroidectomy leaving bilateral remnants of < 1g on each side is assumed to relevantly reduce the incidence of transient postoperative hypoparathyroidism with equal therapeutic safety in terms of recurrent disease, deterioration of endocrine orbitopathy and method-associated complications. The trial is likely to optimize the surgical therapy of Graves’ disease. If a lower incidence of transient postoperative hypoparathyroidism is assumed, patient’s safety and quality of life are likely to be enhanced and postoperative care and treatment will be simplified. Care and treatment costs are likely to be relevantly reduced.

## Trial status

The trial manuscript has been reviewed by the Study Center of the German Surgical Society (SDGC). It has been approved by the National Network for Surgical Trials (CHIR-Net) of the German Society of General and Visceral Surgery (DGAV). The project was approved by the German Society of Endocrine Surgery (CAEK) at their 30th annual meeting on the 26th of November 2011 in Innsbruck, Austria. So far 20 high volume centers assigned a preliminary declaration of commitment with a mean number of 23 estimated patients to be enrolled within 12 months. The local ethics committee of the Philipps-University of Marburg approved the trial giving a positive vote on the 25th of April 2012 (leading ethics committee number 35/12). The trial is registered at the German Clinical Trials Register (DRKS) (trial number DRKS 00004161). Financing is applied within the governmental funding program for clinical trials of the German Ministry for Education and Research (BMBF). The Study team is awaiting BMBF response.

## Abbreviations

AWMF: Work Group of the Scientific Medical Professional Societies; BMBF: German Ministry for Education and Research; Ca: Calcium; CAEK: German Society of Endocrine Surgery; CHIR-Net: National Network for Surgical Trials; DGAV: German Society of General and Visceral Surgery; fT3: Free triiodinethyronine; fT4: Thyroxine; LEMO: Eyelid exophthalmos eye muscle, optic nerve; PTH: Parathyroid hormone; SDG: Study Center of the German Surgical Society; SF36: Clinical Studies 36-Item Medical Outcomes Study Short-Form General Health Survey; TRAK: Thyroidea receptor anti-body; TSH: Thyroid stimulating hormone.

## Competing interests

The authors hereby declare that there are no financial or non-financial competing interests within the conception or conduct of the trial.

## Authors’ contributions

DKB reviewed the recommendation of the national guideline through a systematic PubMed literature search of the current evidence and generated the hypothesis. KM worked out a trial concept, created a study protocol and presented and discussed the idea within the national surgical societies (DGAV, and CAEK) and clinical trials boards (SDGC, and CHIR-Net). She applied for BMBF-funding, let the study be registered and wrote the present paper for TRIALS publication. DL carried out all statistical calculations and outlined the biometric concept. KS and CN substantially contributed to the definition and form of the trial concept and critically reviewed the trial protocol. All authors read and approved the final manuscript.
